# Single-stranded DNA (ssDNA) donor repair templates and CRISPR/Cas9 enable a high-frequency of targeted insertions in potato

**DOI:** 10.3389/fgeed.2025.1661829

**Published:** 2025-09-19

**Authors:** Matías N. González, Neha Salaria, Martin Friberg, Ying Liu, Josefin Alverup, Mariette Andersson, Per Hofvander

**Affiliations:** Department of Plant Breeding, Swedish University of Agricultural Sciences, Lomma, Sweden

**Keywords:** gene editing, CRISPR, DRT, HDR, homologous recombination, protoplast, MMEJ

## Abstract

Homology-directed repair (HDR) holds great promise for plant genetic engineering but remains challenging due to its inherently low efficiency in gene editing applications. While studies in animal systems suggest that the structure of the donor repair template (DRT) influences HDR efficiency, this parameter remains largely unexplored in plants. In this study, we combined protoplast transfection with next-generation sequencing to analyse the impact of DRT structure on HDR efficiency in potato. A highly efficient ribonucleoprotein (RNP) complex targeting the *soluble starch synthase 1* (*SS1*) gene was used in combination with various DRTs, differing in structural factors such as homology arm (HA) length, strandedness (i.e., ssDNA vs. dsDNA), and sequence orientation in ssDNA donors. Our results indicate that a ssDNA donor in the target orientation outperformed other configurations, achieving a HDR efficiency of 1.12% of the sequencing reads in the pool of protoplasts. Interestingly, HDR efficiency appeared independent of HA length. Notably, a ssDNA donor with HAs as short as 30 nucleotides led to targeted insertions in up to 24.89% of reads on average, but predominantly via alternative imprecise repair pathways, such as microhomology-mediated end joining (MMEJ). This donor structure also consistently yielded the highest HDR and targeted insertion rates at two out of three additional loci tested, offering valuable insights for future genome editing strategies in potato. We further assessed strategies to favour HDR over alternative repair outcomes, including the use of small molecules known to inhibit competing pathways in animal systems, and modifications to DRTs to enhance their availability in the vicinity of the target site. However, these approaches did not improve HDR efficiency. Overall, this study presents an effective platform for rapidly assessing gene editing components in potato and provides insights for achieving high-frequency, targeted insertions of short DNA fragments, especially relevant for efficient knock-in integration in non-coding genomic regions.

## 1 Introduction

Potato (*Solanum tuberosum* L.) is among the world’s most important food crops. Beyond serving as a vital energy source, potato tubers provide essential nutrients, including vitamins B and C, phenolic compounds, minerals, and high-quality protein, reinforcing their significance for global food security (Burgos et al., 2020; Devaux et al., 2020). Additionally, potatoes are a key source of starch, a renewable bulk product widely used in food and industrial applications (Dupuis and Liu, 2019). However, potato cultivation requires high inputs to manage abiotic and biotic stresses, challenges exacerbated by climate change. Traditional breeding methods, while effective, are time-consuming and complicated by potato complex genetics, characterized by a tetraploid inheritance, high heterozygosity, and inbreeding depression (Bonierbale et al., 2020).

Recent advances in genome editing, particularly the clustered regularly interspaced short palindromic repeat (CRISPR) system, offer precise and efficient tools for targeted genetic modifications. The CRISPR/Cas9 system (Jinek et al., 2012) is established as the most widely used gene-editing platform due to its simplicity, efficiency, and cost-effectiveness (Zhu et al., 2020; Gao, 2021; Cardi et al., 2023). In its simplest application, CRISPR/Cas9 facilitates targeted mutagenesis via a single guide RNA (sgRNA) that directs the Cas9 nuclease to a specific genomic site, where it induces a double-stranded break (DSB). This type of break is primarily repaired through the error-prone non-homologous end joining (NHEJ) pathway (Puchta, 2005), leading to small insertions or deletions (indels). Alternatively, the presence of short regions with homology (microhomologies) flanking the Cas9-induced DSB can trigger the microhomology-mediated end joining (MMEJ) pathway, resulting in larger deletions whose size is determined by the distance between the microhomologies (Sfeir et al., 2024). Targeted mutagenesis has a prominent role for studying gene functions and engineering commercially valuable traits in several crops, including potato (Hofvander et al., 2022; Tuncel and Qi, 2022).

However, many agronomically important traits require precise modifications in coding or regulatory regions rather than simple gene knockouts (Gilbertson et al., 2025). When a donor repair template (DRT) is available, the homology-directed repair (HDR) pathway can be activated, enabling precise insertions or substitutions. The DRT molecule is designed with a desired insert and flanking homology arms (HAs) that facilitate the incorporation of edits into the genome. HDR-mediated gene editing has been demonstrated in different plant species, including potato, though at a low frequency of precise recombination events (Butler et al., 2016; Hegde et al., 2021). The reason is that HDR is typically infrequent in somatic plant cells, as it is limited to the S and G2 phases of the cell cycle, unlike the most frequent NHEJ, which operates throughout the entire cycle (Schmidt et al., 2019). Additionally, inefficient DSB induction and poor DRT availability near the DSB further constrains HDR activation (Cermak, 2021).

Given its potential in crop improvement, increasing HDR efficiency in plant species remains a critical research focus. Strategies to enhance HDR include promoting conditions that favour HDR over NHEJ and increasing local DRT availability (Chen et al., 2022; Singh et al., 2023). While various methods to boost HDR frequencies have been tested in animal systems (Singh et al., 2023), studies in plants remain limited (Cermak, 2021).

Research in animal models suggest that DRT structure significantly influences HDR activation. Key factors include HA length, the ratio between HA and insert fragments, the strandedness of the DRT molecule (single-stranded [ss] vs. double-stranded [ds] DNA), and sequence orientation in ssDNA molecules (Baker et al., 2017; Quadros et al., 2017; Miura et al., 2018; Bai et al., 2020; Ranawakage et al., 2020). For example, a systematic evaluation in mice demonstrated that for dsDNA donors, HDR efficiency increases sharply as HAs extend from 200 bp to 2,000 bp, with more moderate gains observed for HAs longer than 2,000 bp and up to 10,000 bp (Baker et al., 2017). In human cells, Zhang et al. (2017) reported a similar trend, with HDR efficiency gradually increasing as HAs extended from 50 bp to 900 bp, although sequences as short as 50 bp still enabled 6%–10% HDR efficiency (Zhang et al., 2017). Regarding the use of ssDNA as donors, high HDR efficiency appears achievable even with short HAs. For instance, in mice, combining ssDNA DRTs with ribonucleoprotein (RNP) delivery of editing components resulted in HDR efficiencies ranging from 8.5% to 100% for HAs of 50–100 nucleotides, even for large inserts (>800 bases) (Quadros et al., 2017; Miura et al., 2018). Similar findings were reported in zebrafish, where ssDNA outperformed dsDNA for HDR-mediated editing, even with 40 nucleotides-HAs (Bai et al., 2020; Ranawakage et al., 2020).

DRTs as ssDNA molecules can be used in one of two possible orientations relative to the sgRNA recognition sequence. The “target” orientation coincides with the strand that is recognised by the sgRNA, whereas the “non-target” orientation corresponds to the opposite strand containing the PAM sequence. While some studies in animals have indicated that there may be a preference for using either orientation (Paix et al., 2017; Skarnes et al., 2019), other studies demonstrate that the optimal orientation may be dependent on the target locus and its sequence (Ranawakage et al., 2020). Thus far, no empirical analysis of this parameter for specific target sites and donor molecules have been conducted in plants.

Despite extensive research conducted in animal models, research on DRT structure and its impact on HDR efficiency in plants remains limited. Jiang et al. (2021) studied how HA length influences HDR efficiency in *Nicotiana benthamiana* protoplasts transfected with CRISPR/Cas9 RNPs, finding that HAs longer than ∼35 and up to ∼64 nucleotides achieved highest HDR efficiency (∼45%) in the pool of protoplasts (Jiang et al., 2021). However, all other parameters related to DRT structure were kept invariable. Given the lack of established guidelines for optimal DRT design, this study investigates how DRT structure influences HDR efficiency in potato. To this end, we employed ribonucleoprotein (RNP)/DRT transfections in potato protoplasts, combined with Next-generation sequencing (NGS), to precisely quantify editing outcomes. Our results demonstrate that DRT structure significantly impacts HDR efficiency, with ssDNA donors in the target orientation outperforming other structures at three of the four tested genomic loci. In contrast, the length of HAs appeared to have a comparatively minor effect on HDR efficiency, within the tested range of 30–97 nucleotides. Efforts to enhance HDR by modulating DNA repair pathways or increasing DRT availability, using strategies commonly successful in animal systems, did not improve outcomes. Furthermore, sequencing revealed a high-frequency of targeted insertions, likely driven by alternative repair mechanisms, highlighting the potential for efficient knock-in integration at non-coding regions in the potato genome.

## 2 Results

### 2.1 On-target cleavage efficiency varies with target site selection

**FIGURE 1:**
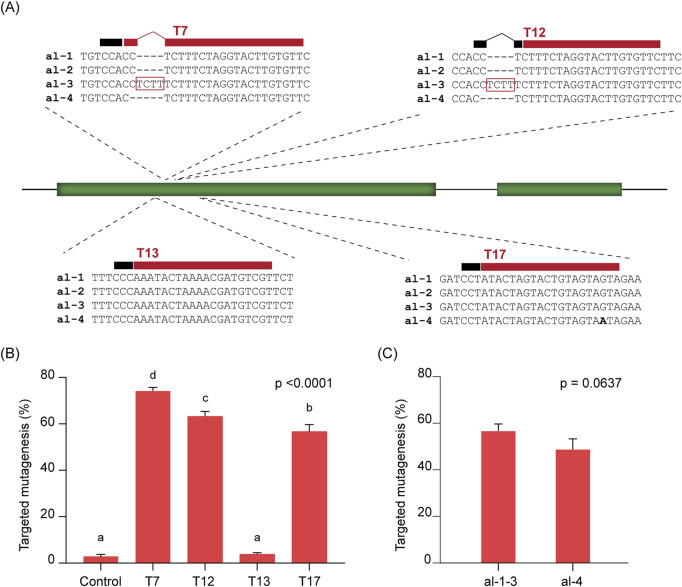
On-target cleavage efficiency at the *SS1* gene. **(A)** Schematic representation of the *SS1* gene and selected target sites T7, T12, T13, and T17. The different alleles are aligned in each case. The 20-nucleotide target sequences are indicated by red bars above alignments, and the position of the PAM is shown as a black bar. **(B)** Targeted mutagenesis (%) observed at each target site and at a negative control. For target sites with allelic variation (T7, T12, and T17), only reads matching the corresponding target allele were considered. ANOVA’s p-value is indicated. Different letters denote statistically significant differences determined using Tukey’s multiple comparisons test (p < 0.05). **(C)** Targeted mutagenesis (%) at different alleles of the T17 target site. No significant differences were found between alleles, according to an unpaired t-test (p-value is indicated). In **(B,C)**, data is presented as the mean of three independent biological replicates with SD error bars.

The first step for an effective HDR strategy is the induction of a DSB at the target site. Since higher frequencies of DSBs can lead to increased HDR efficiency (Puchta et al., 1996), and DSB induction efficiency depends largely on the choice of sgRNA, we tested four sgRNAs targeting different sites within the *soluble starch synthase 1* (*SS1*) gene in potato cultivar Kuras (Figure 1A).

As Kuras is a tetraploid cultivar carrying four alleles of each locus, the target site designated T13 is conserved across all four *SS1* alleles. In contrast, the targets T7 and T12 are found in two out of the four alleles, while T17 is present in three alleles (Figure 1A). We assessed the efficiency of each sgRNA by protoplast transfection followed by NGS and quantification of mutagenesis frequency, focusing only on those alleles containing the respective target site (Figure 1B). As expected, targeted mutagenesis efficiency varied among the different sgRNAs. The highest average frequency was observed for T7 (74.19%), followed by T12 (63.26%) and T17 (56.73%). In contrast, T13 showed a much lower average mutagenesis frequency (3.82%), which was not significantly different from the negative (mock-transfected) control (2.92%).

The high allelic variation observed at the most efficient target sites, T7 and T12, prevented mutagenesis on the alternate alleles due to multiple mismatches near the PAM in T7 (alleles 3 and 4, Figure 1A), and the absence of a PAM in T12 (alleles 3 and 4, Figure 1A). However, for T17, the remaining alternate allele (allele 4, Figure 1A) contains only a single mismatch located distal to the PAM. Although a slight reduction in mutagenesis frequency was observed (48.76%), there was no statistically significant difference in targeted mutagenesis between the alleles at T17 (Figure 1C). This result indicates that the sgRNA designed for T17 is broadly effective on both types of *SS1* alleles, inducing DSBs with frequencies ranging from 48.76% to 56.73%. Based on these findings, the sgRNA targeting T17 was selected for further analysis in our study.

Since targeted mutagenesis can arise from non-homologous end joining (NHEJ) and/or the microhomology-mediated end joining (MMEJ) mechanisms, we analysed the incidence of highly-predicted MMEJ-patterns in mutational outcomes at T17 (Supplementary Table S1). At least 21.51% of all mutational patterns obtained were consistent with MMEJ (Supplementary Table S1).

### 2.2 Donor repair template (DRT) structure influences HDR efficiency

**FIGURE 2:**
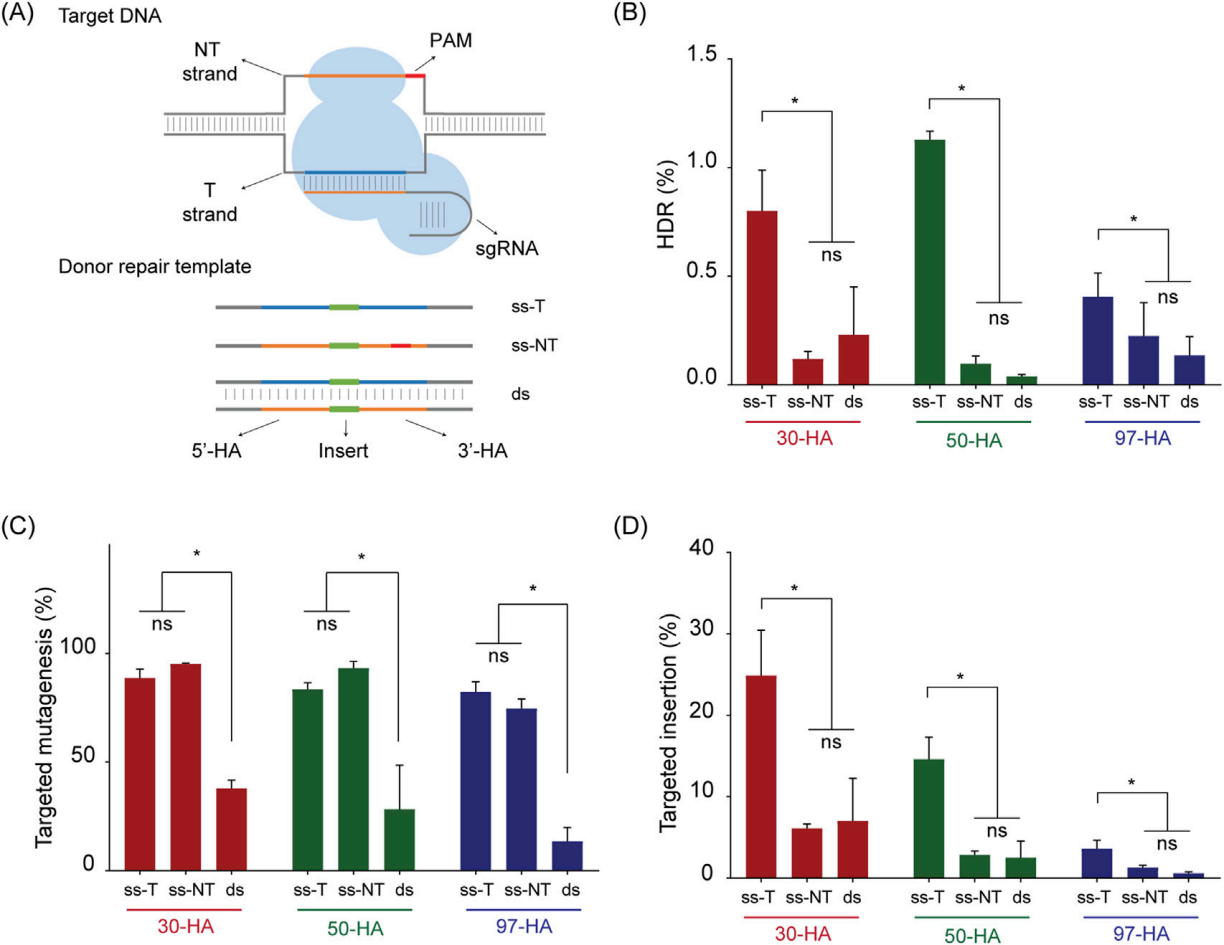
Donor repair template (DRT) structure on *SS1* gene editing. **(A)** Schematic representation of the target DNA and Donor Repair Template used for transfections. In target DNA, the target strand “T-strand” (represented as blue line) is complementary to the sgRNA sequence, whereas the non-target strand (“NT strand”, orange line) contains the 20 nucleotides sequence included in the sgRNA and the PAM (represented as a red line). In Donor Repair Template, the position of the insert is shown as a green bar, flanked by homology arms (5′ HA and 3′ HA). Blue and orange colours are used to represent sequences matching with the target site strands, and PAM is represented in red. **(B)** Homology-directed repair (HDR) efficiency (%) achieved with each DRT. **(C)** Targeted mutagenesis (%) at the T17 target site. **(D)** Targeted insertions (%) of the *BamHI* recognition site for each DRT. “ss-T,” “ss-NT,” and “ds” indicate ssDNA in the target orientation, ssDNA in the non-target orientation, and dsDNA, respectively. Homology arm lengths are indicated as 30-HA, 50-HA, and 97-HA. Data is presented as the mean of three independent biological replicates and error bars for SD. Statistically significant differences, determined by Tukey’s multiple comparisons test (p < 0.05), are marked with asterisks; “ns” indicates no significant difference.

In animal models, HA length, the number of DNA strands in the donor molecule, and the sequence orientation of ssDNA molecules are known to influence the HDR efficiency (Schubert et al., 2021). To assess the relevance of these factors in potato, we designed nine different DRTs to insert a six-base-pair *BamHI* restriction site (5′-GGATCC-3′) at the T17 target site (Figure 2A). The insert was flanked by HAs of 30, 50, or 97 nucleotides, and each donor was tested as either dsDNA or ssDNA, with the later provided in either the target (ss-T) or non-target (ss-NT) strand orientation (Figure 2A).

We used protoplasts transfection and NGS analysis to determine the effects of HA length and strand selection. Notably, the use of ss-T consistently outperformed the other donor types across all HA lengths, with mean HDR efficiencies ranging from 0.40% to 1.12% of total analysed reads (Figure 2B). A two-way ANOVA revealed that HA length had no significant effect on HDR efficiency, whereas donor strand selection had a highly significant effect (Table 1).

**TABLE 1:** Two-way ANOVA analysis DRT structure, with simple main effect analysis.

Source	Type III Sum of Squares	df	Mean Square	F (DFn, DFd)	P value
HDR efficiency (%)					
Strand selection	2.436	2	1.218	F (2, 22) = 17.66	P < 0.0001
HA length	0.1367	2	0.06834	F (2, 22) = 0.9910	P = 0.3872
Residual	1.517	22	0.06896		
Targeted insertion (%)					
Strand selection	720.5	2	360.2	F (2, 22) = 20.19	P < 0.0001
HA length	530	2	265	F (2, 22) = 14.86	P < 0.0001
Residual	392.5	22	17.84		

Variations in overall CRISPR/Cas9 activity could explain the differences in HDR efficiency with the various donors. To investigate this, we quantified the frequency of targeted mutagenesis across our dataset (Figure 2C). Mutagenesis frequencies were statistically identical in experiments using ss-T and ss-NT donors but were significantly reduced in experiments employing dsDNA donors (Figure 2C). For instance, in DRTs carrying 30 nucleotides-HAs, the mean targeted mutagenesis was 88.75% and 95.15% for ss-T and ss-NT, respectively, and 37.88% for the dsDNA molecule. Similar significant reductions in targeted mutagenesis were found for dsDNA molecules containing 50 and 97 nucleotides-HAs (Figure 2C).

When a DRT is available, insertions can also occur via alternative repair pathways such as NHEJ or MMEJ (Hsu et al., 2021; Van Vu et al., 2021; Kumar et al., 2023; Vu et al., 2024). To explore this, we quantified the frequency of reads containing the desired insert in the correct orientation, irrespective of perfect recombination at the flanking HAs. We refer to these events as “targeted insertions” throughout the manuscript. The pattern of targeted insertions mirrored that of HDR, with ss-T donors producing the highest insertion frequencies across all HA lengths (Figure 2D). Interestingly, a two-way ANOVA indicated that both HA length and strand selection significantly influenced targeted insertion frequency (Table 1). Our results showed that shorter HAs (30 nucleotides) resulted in the highest frequency of targeted insertions, followed by 50 and then 97 nucleotides-HAs (Figure 2D). Notably, the ss-T donor carrying 30 nucleotides-HAs yielded the highest targeted insertion frequency, reaching 24.89% of total reads (Figure 2D). This represents a 31-fold increase compared to the HDR efficiency achieved with the same donor.

Targeted insertions derived from NHEJ and MMEJ can often be distinguished by size distribution (Kumar et al., 2023). To further characterise the high-frequency insertions obtained with our ss-T donor carrying 30-nucleotide HAs, we analysed insertion sizes and profiles across the sequencing data (Supplementary Figure S1). The vast majority of insertions were 10–30 nt in length, primarily resulting from partial duplications of the 5′ HA at the target site (Supplementary Figure S1). In contrast, analysis of the equivalent dsDNA donor revealed additional, larger insertions (≥60 nt), caused by complete duplication of both HAs as well as reversely oriented duplications (Supplementary Figure S1).

In summary, our analysis demonstrates that the structure of the DRT significantly influences HDR efficiency, with ssDNA in the target strand orientation outperforming all other structures. Moreover, targeted insertions mediated by alternative repair mechanisms occur at much higher frequencies than HDR in potato, and seem to be favoured by inclusion of shorter HAs.

### 2.3 Chemical inhibitors do not affect non-homologous end joining (NHEJ)

Given the marked difference between HDR efficiency and the frequency of reads containing targeted insertions, we next evaluated a panel of small molecules reported to inhibit NHEJ in animal systems, aiming to determine whether blocking this alternative repair pathway could enhance the frequency of precise insertions mediated by HDR. The inhibitors tested included the histone deacetylase (HDAC) inhibitor Trichostatin A (TSA) (Robert et al., 2016), the DNA-dependent protein kinase catalytic subunit (DNA-PKcs) inhibitor NU7441 (Leahy et al., 2004), and the Alt-R HDR Enhancer V2 (Integrated DNA Technologies, Inc.) (Kath et al., 2022; Shy et al., 2023).

**FIGURE 3:**
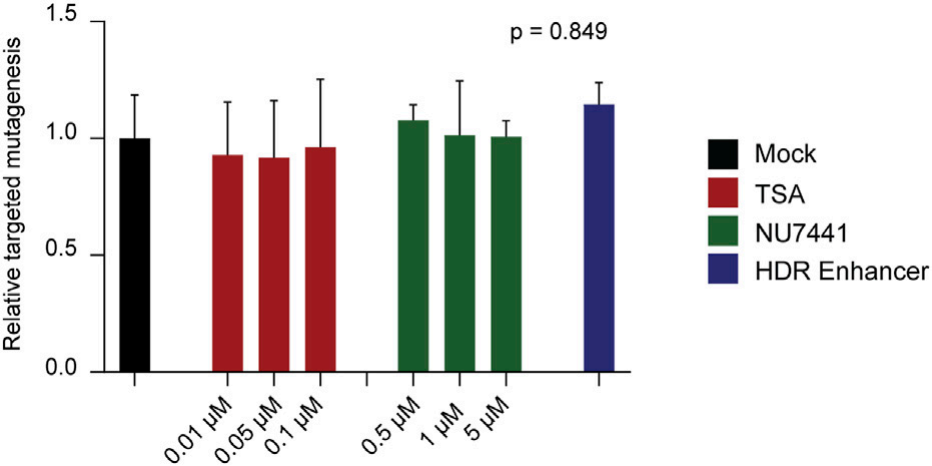
Use of non-homologous end joining (NHEJ) inhibitors in potato protoplasts. Effect of Trichostatin A (TSA), NU7441, and Alt-R HDR Enhancer on targeted mutagenesis at the T17 target site. The final concentrations of TSA and NU7441 in the culture medium are indicated. Data is presented as the mean of three independent biological replicates, with SD error bars. Values were normalized to the mean of the mock control. ANOVA’s p-value is indicated.

Using our most efficient donor construct, ss-T carrying 30 nucleotides-HAs, we transfected potato protoplasts and incubated them for 48 h in growth medium supplemented with each of the inhibitors. We first evaluated whether NHEJ activity was affected by the different inhibitors, by quantifying the frequency of reads showing targeted mutagenesis. Contrary to expectations, no significant differences in targeted mutagenesis were observed between inhibitor-treated and control protoplasts (Figure 3). In addition, to rule out potential changes in mutational profile, we further analysed the incidence of MMEJ-compatible mutations in our dataset. Again, no differences were detected between inhibitor-treated and control protoplasts (Supplementary Figure S2). In agreement with these results, no positive effects were observed in HDR efficiency nor in targeted insertions across treatments (Supplementary Figure S2).

These results indicate that under our experimental conditions, the tested inhibitors did not suppress NHEJ and, consequently, did not enhance the frequency of HDR.

### 2.4 Incorporation of truncated Cas9 target sites (CTS) reduces HDR efficiency in potato

Enhancing the availability of the DRT for the cellular repair machinery represents a viable strategy to improve HDR efficiency. In human cells, HDR has been successfully enhanced by incorporating truncated Cas9 target sites (CTS) at both ends of the DRT (Nguyen et al., 2020; Shy et al., 2023). These truncated sites are recognized by Cas9, promoting the formation of RNP-DRT complex and facilitating co-localization within the nucleus, thereby increasing the likelihood of HDR-mediated repair (Nguyen et al., 2020; Shy et al., 2023). CTS-DRTs can be delivered either as complete dsDNA molecules (CTS-ds) (Nguyen et al., 2020) or as a hybrid molecules, composed of a ssDNA donor with short regions of dsDNA containing the CTS on each flank (CTS-ss) (Shy et al., 2023).

**FIGURE 4:**
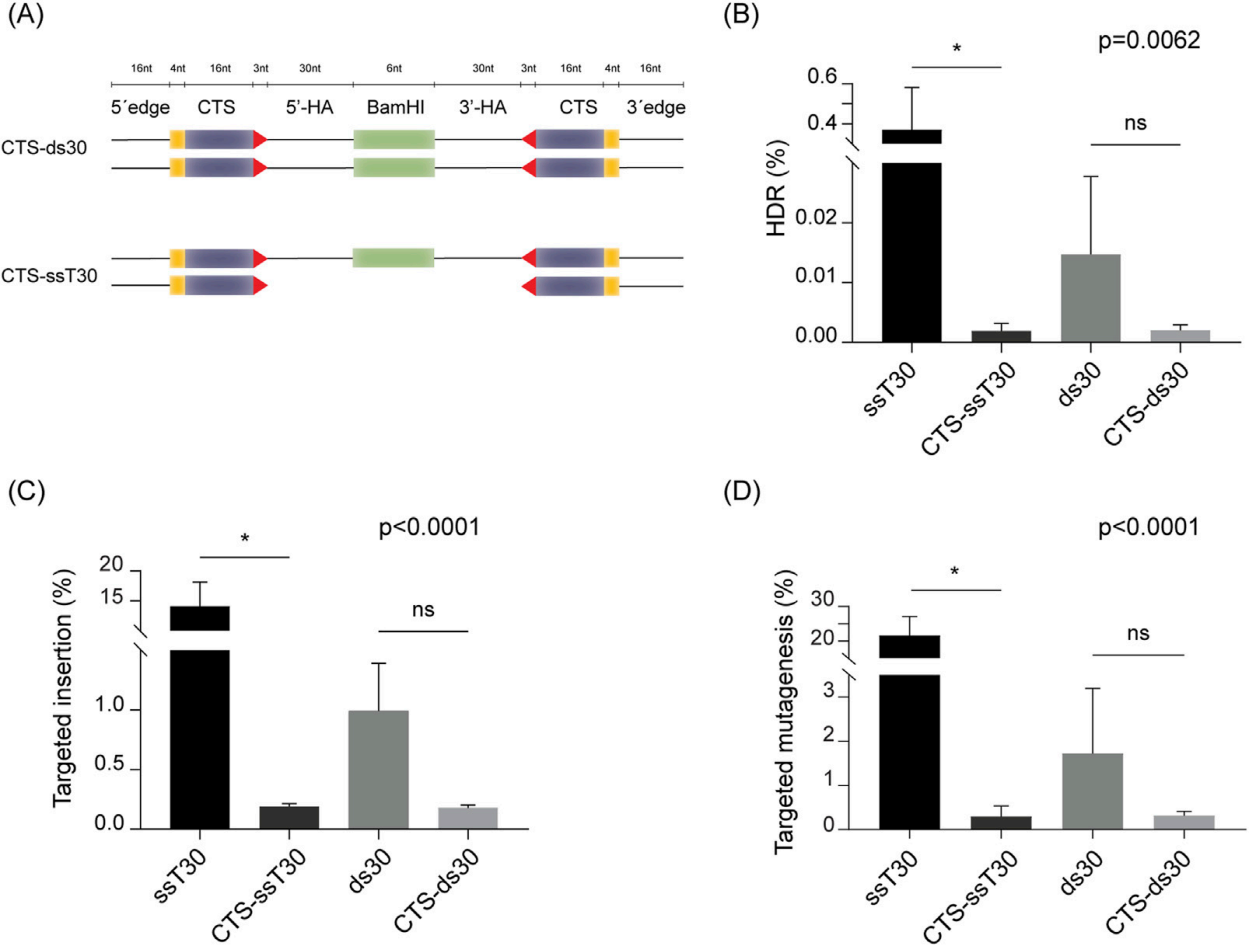
Incorporation of Cas9 target sites (CTS) into donor molecules. **(A)** Schematic representation of the truncated Cas9 target sites (CTS)-containing donor molecules. Length of each element is expressed as number of nucleotides (nt) above the illustrations. Truncated (16 nt) Cas9 target sites are represented as purple bars with the four mismatched nucleotides represented as yellow bars, and the PAM position and orientation indicated as a red arrowhead. “5′-HA” and “3′-HA” indicate the 30 nt-homology arms flanking the *BamHI* recognition insert (indicated as a green box). Additional 16 nt-sequence of DNA edges was added to the 5′ and 3′ ends, following the description in (Nguyen et al., 2020). **(B)** HDR efficiency (%) achieved with each donor. **(C)** Targeted insertion (%) of the *BamHI* recognition site for each DRT. **(D)** Targeted mutagenesis (%) at the T17 target site. Data is presented as the mean of three independent biological replicates, with SD error bars. ANOVA’s p-value are indicated on each graph. Statistically significant differences, determined by Sidak’s multiple comparisons test (p < 0.05), are marked with asterisks; “ns” indicates no significant difference.

To assess this strategy in potato, we incorporated CTS at both ends of our most efficient donor carrying 30-nucleotide homology arms, to create a dsDNA (CTS-ds30) and a hybrid (CTS-ssT30) donor. Details on the structure of the employed CTS-DRTs are shown in Figure 4A. Each donor was assessed in protoplasts, and compared to the respective control carrying no CTS. The use of the hybrid CTS-ssT30 resulted in a HDR efficiency of 0.0019%, significantly lower than that of the control (0.3706%) (Figure 4B). Similar reductions were obtained in targeted insertion, with CTS-ssT30 resulting in 0.19% targeted insertion, significantly lower than the 14.11% obtained with the control (Figure 4C). Despite no statistically significant differences, the same trend was observed for HDR efficiency and targeted insertion between CTS-ds30 and the corresponding control with no CTS (Figures 4B,C).

Furthermore, analysis of targeted mutagenesis frequencies showed consistent reductions upon CTS inclusion, indicating an overall decline in CRISPR/Cas9 activity (Figure 4D). The results mirrored those of the HDR efficiency and targeted insertion, with use of CTS-ssT30 showing a significant reduction in targeted mutagenesis related to the control, and CTS-ds30 displaying a marked lower targeted mutagenesis, despite no statistically significant differences with its control.

Collectively, these findings suggest that the incorporation of truncated Cas9 target sites into donor molecules, negatively impacts genome editing outcomes in potato. In our system, CTS-DRTs appear to reduce both HDR efficiency and the frequency of targeted insertions, likely due to diminished CRISPR/Cas9 activity.

### 2.5 Target site election influences ssDNA donor-mediated HDR efficiency

Our results targeting the *SS1* locus revealed that ssDNA donors lead to higher HDR efficiency, with a clear preference for donors oriented as the target strand (ss-T donors) rather than those oriented as the non-target strand (ss-NT donors). To further explore the generality of this observation, we targeted three additional loci in potato: *EID1* (empfindlicher im dunkelroten licht 1), *LNK2* (night light–inducible and clock-regulated gene 2), and *SES* (suppressor of SP6A expression).

**FIGURE 5:**
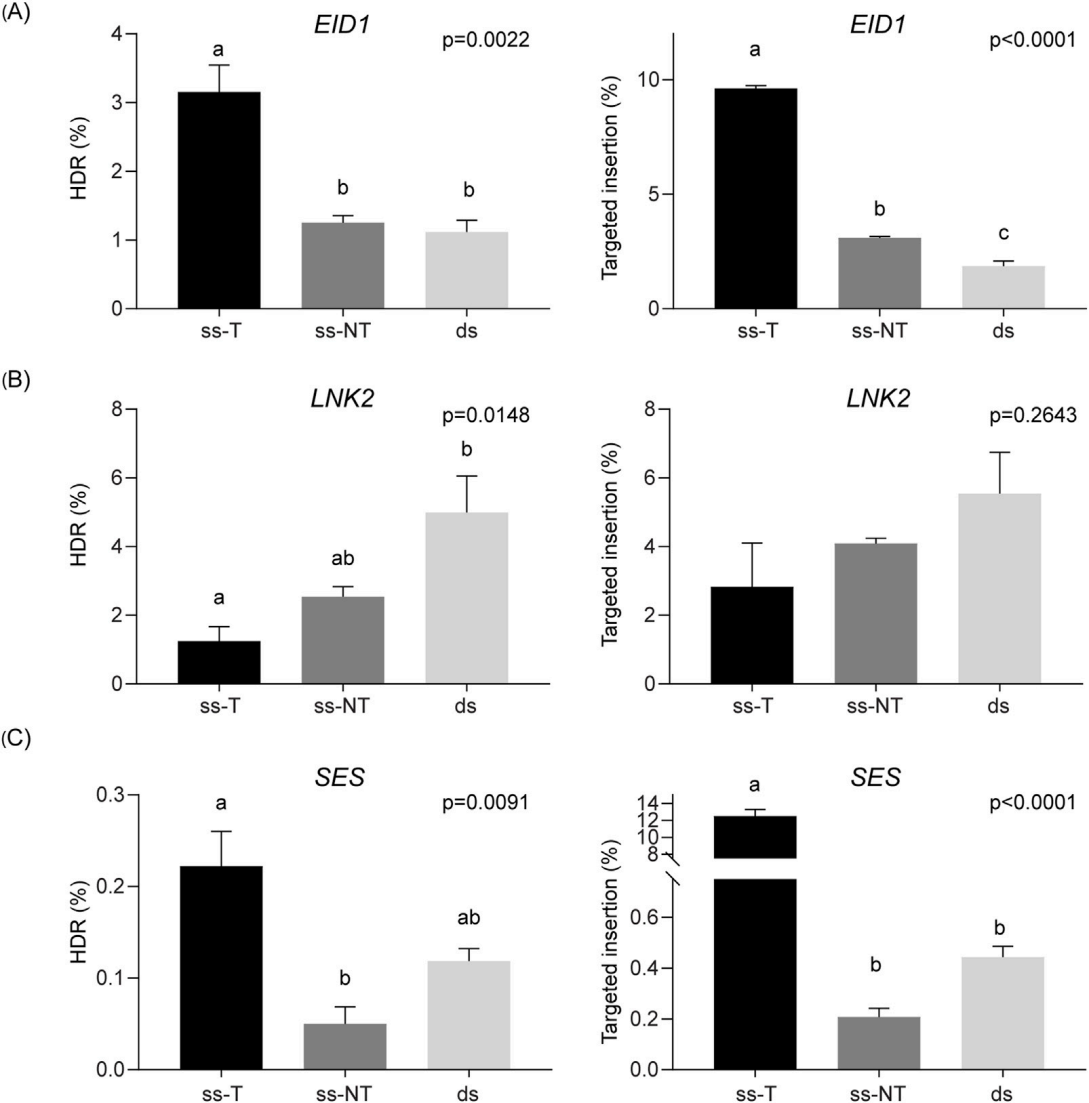
Strand preference for DRTs at different loci. HDR efficiency (%) and targeted insertions (%) achieved with each DRT at the *EID1* **(A)**, *LNK2* **(B)**, and *SES* **(C)** target genes. Data is presented as the mean of three independent biological replicates, and error bars indicate SD. ANOVA’s p values are indicated on each graph and different letters denote statistically significant differences determined using Tukey’s multiple comparisons test (p < 0.05).

Highly efficient sgRNAs were selected for each target gene (Supplementary Figure S3), and DRTs were designed with a *BamHI* restriction site as insert, flanked by 30 nucleotides-HAs. At both *EID1* and *SES*, our findings were consistent with those observed for *SS1*, with the highest HDR efficiencies obtained using ss-T donors (3.15% for *EID1* and 0.22% for *SES*, respectively; Figures 5A,C). In addition, ss-T donors resulted in higher frequencies of targeted insertions, reaching 9.62% and 12.50% of total reads for *EID1* and *SES*, respectively (Figures 5A,C). In contrast, targeting *LNK2* yielded higher HDR efficiencies when using ss-NT or dsDNA donors, reaching average values of 2.54% and 4.99%, respectively (Figure 5B). However, analysis of targeted insertion frequencies at *LNK2* revealed no significant differences among the three DRT structures (Figure 5B).

To analyse whether variations in overall CRISPR/Cas9 activity could explain the differences in HDR efficiency at the different target sites, we analysed the targeted mutagenesis across our dataset (Supplementary Figure S4). Consistent with our previous analysis in *SS1*, mutagenesis frequencies were systematically lower when using dsDNA donors (Supplementary Figure S4).

Taken together, these results demonstrate that the optimal strand selection for maximizing HDR efficiency is locus-dependent in potato. Nevertheless, in most of the tested loci, ss-T donors consistently led to the highest HDR efficiencies in agreement with the previous results in *SS1*, highlighting a general trend that may inform future genome editing strategies in this species.

## 3 Discussion

Gene editing using the CRISPR/Cas9 system is a powerful tool for genetic engineering and is significantly accelerating the improvement of agronomically important traits in a wide range of crop species. HDR can mediate precise changes, but its efficiency in somatic plant cells remains low (Cermak, 2021). In our study, we investigated conditions with the potential to favour HDR-mediated gene editing in potato using a protoplast transfection system coupled with NGS analysis.

Since the induction of a DSB is an essential initiating step for HDR (Puchta et al., 1996), and HDR efficiency in plants is positively correlated with DSB frequency (Zhang Z. et al., 2022; Li et al., 2024), we began by evaluating four different sgRNAs targeting the *SS1* gene. Editing efficiency varied markedly, ranging from 3.82% for guide T13 (statistically indistinguishable from the mock control) to 74.19% for guide T7 (Figure 1B). These results underscore the well-established impact of sgRNA selection on editing efficiency. Although various bioinformatic tools can predict sgRNA efficacy (Konstantakos et al., 2022), predictions often diverge from experimental outcomes (Concordet and Haeussler, 2018). This observation is even more noticeable in gene editing applications in plants, as most of the efficiency predictors have been trained using empirical data derived from animal models (Naim et al., 2020). Our findings reaffirm that sgRNA performance is best validated empirically, and the protoplast transfection and NGS analysis performed in our study, represent a rapid and reliable approach to this endeavour.

Due to tetraploid nature of the potato variety used in our study and high allelic variability in the *SS1* targeted region, only the sgRNA designed on T13 matched all four alleles (Figure 1A). In contrast, sgRNAs targeting T7 and T12 were not predicted to bind any of the alternate alleles, which was confirmed experimentally (data not shown). The sgRNA designed on T17, however, targeted a region containing a single-nucleotide polymorphism (SNP) in one allele, located distal to the PAM (Figure 1A). Interestingly, sequencing data showed T17 edited all four alleles with comparable efficiency, including the SNP-containing allele (Figure 1C). This supports earlier findings that Cas9 tolerates single mismatches distal to the PAM (Jiang and Doudna, 2017; Feng et al., 2018; Es et al., 2019; Lee et al., 2019; Modrzejewski et al., 2020; Wang et al., 2021), highlighting implications for sgRNA selection in regions with allelic variation and also the prediction of potential off-target effects in highly similar loci.

In addition to efficient DSB induction, HDR also requires the presence of a DRT carrying the desired insert. Although extensively studied in animal models, the impact of DRT structure on HDR efficiency in plants is poorly understood. In a previous report using *N. benthamiana* protoplasts, Jiang et al. (2021) examined how HAs length affects HDR. Other DRT parameters were constant in that study, as ssDNA molecules with target orientation were used in all cases. Furthermore, the efficiency of HDR was determined with a reporter system based on GFP activity restoration upon HDR-mediated editing of its coding sequence (Jiang et al., 2021). While highly valuable for facile and inexpensive analysis of the efficiency of different HDR components, a more thorough analysis through high-throughput sequencing may characterise outcomes more accurately, allowing precise comparisons between different strategies. Our data show that HDR efficiency in potato is influenced by DRT structure, with ssDNA donors matching the target strand orientation (ss-T donors) outperforming other structures in three out of four tested loci (Figures 2B, 5A,C). Notably, for the target site on *SS1*, this was consistent across varying HA lengths, as indicated by our two-way ANOVA results (Table 1). This result indicates that for ss-T donors, HAs as short as 30 nucleotides are sufficient to mediate HDR, consistent with the findings reported in *N. benthamiana* (Jiang et al., 2021). However, a limitation of our study is the fixed insert length, leaving open the question of how the ratio between 30 nucleotides-HAs and insert fragments may impact HDR efficiency.

ssDNA donors are generally more effective than dsDNA in various animal systems, such as zebrafish (Bai et al., 2020) and mammalian cells (Yeh et al., 2019; Zhang X. et al., 2022; Jin et al., 2025). The increased efficiency is often attributed to synthesis-dependent strand annealing (SDSA) mechanism, which requires only short homologous sequences (30–40 nucleotides) to trigger precise insertions (Paix et al., 2017; Jin et al., 2025). In contrast, HDR using dsDNA donors typically requires much longer HAs (0.5–1 kb) (Baker et al., 2017; Zhang et al., 2017). It is also generally accepted that ssDNA represents a less cytotoxic cargo than dsDNA for donors in animal cells (Zhang X. et al., 2022). While further investigation is needed to determine if these factors influence ssDNA performance in potato, our data indicate a consistent reduction in targeted mutagenesis when dsDNA donors were included in the transfection (Figure 2C; Supplementary Figure S4). This observation could be explained by two non-exclusive scenarios. First, the presence of dsDNA donors might reduce overall CRISPR/Cas9 activity. A lower rate of DSBs would directly decrease HDR efficiency in these conditions. One possible explanation is an *in vitro* interaction between the RNP complex and dsDNA donors before transfection, which may interfere with target DNA interrogation in the cell. To test this hypothesis, delivering the CRISPR/Cas9 components via vector-based expression could help to prevent potential pre-transfection interactions. Alternatively, reduced targeted mutagenesis could result from dsDNA-associated cytotoxicity, as widely reported in animal systems (Nguyen et al., 2020; Zhang X. et al., 2022; Shy et al., 2023). This hypothesis could be evaluated by assessing protoplast viability prior to PCR amplification of the target locus. These scenarios assume that variations in transfection efficiencies related to specific donors can be disregarded in our system.

Regarding ssDNA strand orientation, ss-T donors were generally more effective in our study, consistent with what has been previously proposed based on Cas9 cutting dynamics (Jiang and Doudna, 2017). Multiple studies using biochemical, structural, and single-molecule approaches support that the non-target strand is typically cut and released first, which would make it more accessible for interaction with a complementary ssDNA donor for HDR (Richardson et al., 2016; Paix et al., 2017; Wang et al., 2018; Wang et al., 2023). Our data support this, with ss-T donors outperforming in three out of four loci (Figures 2B, 5A,C). Importantly, these differences were not merely due to overall CRISPR/Cas9 activity, as for *SS1* similar frequencies of targeted mutagenesis were observed for ss-T and ss-NT donors (Figure 2C), while for *EID1* and *SES* genes, targeted mutagenesis was even higher in experiments using ss-NT donors (Supplementary Figure S4). Nevertheless, inconsistent strand preference at the *LNK2* target site suggests this may not be a generalised rule, aligning with some observations in animal systems (Ranawakage et al., 2020; Schubert et al., 2021).

When a DRT is available, insertions can also occur via non-HDR pathways such as NHEJ or MMEJ (Hsu et al., 2021; Van Vu et al., 2021; Kumar et al., 2023; Vu et al., 2024). Our analysis of reads containing the desired insert, regardless of perfect HA recombination (“targeted insertions”), indicates these events are relatively frequent outcomes in the potato genome. Using ss-T donors with 30-nt HAs, we observed average targeted insertion frequencies of 24.89%, 9.62%, 2.83%, and 12.50% for *SS1*, *EID1*, *LNK2*, and *SES*, respectively (Figures 2D, 5A–C), while corresponding HDR frequencies were much lower (0.80%, 3.15%, 1.25% and 0.22%, respectively). Similar trends were seen with ss-NT and dsDNA donors. Furthermore, our analysis targeting *SS1* suggest that targeted insertions frequency increases as the length of the donor molecules decreases (Figure 2D). Although our method for quantifying targeted insertions includes HDR-mediated events (see Section 5.7), the difference between values of the two parameters determined for each target site, provides a clear indication that most of the insertions obtained were mediated by imprecise or alternative repair mechanisms, rather than by perfect recombination repairs. High-frequency targeted insertions using ssDNA donors have also been reported in *N. benthamiana* protoplasts (Hsu et al., 2021). By using ss-NT donors, the authors reported targeted insertions reaching frequencies of 10.5%–13.6%, based on single-cell genotype analysis (Hsu et al., 2021). Furthermore, plant regeneration conducted on the transfected protoplasts resulted in 29.3%–31.8% of plants carrying targeted insertions, while only one regenerated plant (8.3% of the total analysed), displayed a precise insertion mediated by HDR (Hsu et al., 2021).

Determining the exact repair mechanism underlying the high-frequency targeted insertion of ss-T donors, is beyond the scope of this study. However, our analysis on the *SS1* target gene suggests compatibility with the MMEJ pathway (Sfeir et al., 2024). Features of this pathway include the presence of microhomologies at deletion junctions following DSB induction, which result in deletion patterns “guided” by these microhomologies, as well as insertions generated through DNA synthesis using short homologous sequences as templates, albeit with less fidelity than HDR (Schmidt et al., 2019; Van Vu et al., 2021; Sfeir et al., 2024). Furthermore, high-frequency insertions mediated by MMEJ have been reported in plants (Schmidt et al., 2019). Two lines of evidence from our study support the involvement of MMEJ in mediating targeted insertions of ss-T donor with 30-nt HAs in *SS1*. First, MMEJ-compatible mutations (i.e., deletions flanked by microhomologies) were observed in a significant fraction of all mutated reads across experiments targeting the T17 site in *SS1* (Supplementary Table S1; Supplementary Figure S2). This suggests MMEJ could be actively repairing DSBs in potato, as observed in other plant species (Tan et al., 2020; Weiss et al., 2020)*.* Second, transfections including the ss-T donor predominantly yielded insertions shorter (10–30 nt) than the full donor length (66 nt) due to partial duplications of the flanking homology arms (Supplementary Figure S1). This outcome is compatible with imprecise base pairing between short HA regions and the target site, followed by DNA synthesis, leading to incomplete donor incorporation at the target locus (Sfeir et al., 2024). In contrast, analysis of the equivalent dsDNA donor revealed higher prevalence of insertion lengths closer to the full donor sequence, suggesting that the majority of these events could be mediated by the NHEJ pathway (Supplementary Figure S1). Likewise, in *Setaria viridis*, dsDNA short donors led to up to 51.1% targeted insertions in protoplasts, whose sizes matched a complete HAs duplication, pointing out at a vast majority being mediated by the NHEJ mechanism (Kumar et al., 2023). However, inferring exact mechanisms based solely on editing outcomes remains challenging and further research efforts are needed to determine precise molecular mechanisms behind targeted insertions observed in our study, possibly using plants defective in specific pathways (Schmidt et al., 2019).

To address the discrepancy between HDR and targeted insertions, we tested chemical inhibition of NHEJ in potato protoplasts, as a strategy to boost HDR in the *SS1* locus. Contrary to genetic suppression of NHEJ (Cermak, 2021; Chen et al., 2022), chemical modulation of repair mechanisms has not been extensively studied in plants. One of tested molecules, NU7441, has been widely used in animal systems to inhibit DNA-dependent protein kinases (DNA-PKcs) that act in the canonical NHEJ mechanism, leading to increased HDR (Robert et al., 2015; Schimmel et al., 2023; Shy et al., 2023). Even though no plant homologs of DNA-PKs have been described (Schmidt et al., 2019), NU7441 was previously applied at a concentration of 1 µM during tomato callus regeneration to enhance HDR efficiency in that species, albeit with moderate effectiveness (Vu et al., 2021). This prior evidence prompted us to test different concentration of NU7441 in our protoplast system. Consistent to the absence of its targeted element in plants, adding NU7441 to the protoplasts culture medium at concentrations of 0.5, 1, or 5 µM did not significantly impact HDR efficiency in the *SS1* locus. Moreover, in agreement with its previous application in tomato (Vu et al., 2021), neither of the tested concentrations resulted in negative impacts on the NHEJ mechanism, represented in our study by the quantification of targeted mutagenesis (Figure 3) and the mutation profile analysis (Supplementary Figure S2). These results suggest that incorporation of NU7441 to the culture medium is not a viable strategy for modulating DNA repair in potato, at least for the evaluated concentrations.

We also tested Alt-R HDR Enhancer V2, generally described as a NHEJ inhibitor (Kath et al., 2022; Shy et al., 2023), and TSA, a histone deacetylase inhibitor (Robert et al., 2016). Alt-R HDR Enhancer V2, effective in animal systems (Kath et al., 2022), had no detectable effect on targeted mutagenesis or HDR efficiency in our protoplast system (Figure 3). Further testing at varying concentrations, other than as employed here following manufacturer’s recommendation, may clarify whether dosage or plant-specific factors are limiting its efficacy. Regarding TSA, although generally described as a NHEJ inhibitor, it has shown mixed effects on DNA repair modulation (Singh et al., 2023). For instance, in human cells TSA interfered with NHEJ mechanism, by inhibiting the deacetylation of key factors, such as Ku70 and Ku80, limiting their access to the DSB (Robert et al., 2016). Additionally, TSA increased the duration of the G2 phase in the cell cycle in animal cells, contributing to higher HDR efficiency (Li et al., 2020; Shy et al., 2023). Conversely, in the absence of donor molecules, TSA increased the targeted mutagenesis in animal cells, possibly due to a higher accessibility of the CRISPR/Cas9 components to due to an open chromatin state at target sites, and activation of alternative end-joining repair mechanisms (Li et al., 2020). Previous to our study, concentrations of 0.1 µM–10 μM TSA, increased targeted mutagenesis rates in lettuce and tobacco protoplasts (Choi et al., 2021), potentially by enhancing RNP access through chromatin relaxation. Here, we tested TSA at various concentrations in potato and in presence of a donor molecule. However, tested concentrations did not significantly influence either targeted mutagenesis or HDR (Figure 3). Since TSA’s impact on genome editing may depend on the basal chromatin state at specific target sites, the higher targeted mutagenesis previously reported in lettuce and tobacco may not be generalizable to all loci. Given its promising use in other systems and observation of higher CRISPR/Cas9 activity in other plant systems, additional investigation of TSA would be relevant in potato. Altogether, our results using chemical molecules with potential modulation effects on DNA repair mechanisms provide evidence that directly translating strategies validated in animal studies remains challenging, possibly due to differences in the components and molecular mechanisms operating in plant systems (Schmidt et al., 2019).

In an attempt to further enhance HDR efficiency, we investigated a strategy to increase local availability of DRT for *SS1* gene editing. To this end, we incorporated truncated Cas9 target sites (CTS) at the ends of the HAs in the donor molecules. This approach has previously been shown to increase HDR efficiency by up to threefold in human cells transfected with dsDNA donors (Nguyen et al., 2020), and has also been applied successfully to ssDNA donors (Shy et al., 2023). In human cells, CTSs comprising 16 bp of the target sequence enable Cas9 to bind, but not cleave, the DRT, thereby enhancing its nuclear localization through co-translocation with the RNP complex. In contrast, the inclusion of CTS at the ends of the 30-nucleotide HAs in the ss-T donor designed for the *SS1* locus significantly reduced HDR efficiency in our potato system (Figure 4A). A similar reduction was observed with the corresponding dsDNA donor, although in this case, the difference compared to the control was not statistically significant (Figure 4A). Based on targeted mutagenesis analysis, we concluded that the inclusion of CTSs reduced overall CRISPR/Cas9 activity (Figure 4D). In previous applications of this strategy in human cells, pre-incubation of the RNP complex with the CTS-containing donor was essential to achieve high HDR efficiency. This may be due to the necessity of forming a stable interaction between the RNP and the CTS-containing DRT, enabling their joint translocation into the nucleus (Nguyen et al., 2020). In our study, such interactions may have interfered with Cas9 activity at the genomic target site in potato, possibly due to competitive binding or steric hindrance. This observation supports our earlier hypothesis regarding potentially deleterious interactions between dsDNA donors and Cas9 prior to protoplast transfection. Additionally, a potential decrease in DNA cleavage efficiency in strategies that tether the donor molecule to the RNP complex, has been suggested by other authors (Jin et al., 2024; Jin et al., 2025). Alternative successful strategies to enhance local DRT availability have been explored in rice, such as the use of RNA donor molecules as extensions of the sgRNA (Butt et al., 2017), or fusion of the *Agrobacterium*-derived VirD2 protein to Cas9, which enables tethering of a ssDNA donor to the editing complex (Ali et al., 2020; Tang et al., 2023). Despite the reduced efficacy observed in our system, the demonstrated success of CTS-based donors in human cells and their compatibility with RNP-based, transgene-free editing strategies suggest that further optimisation of this approach could still hold promise for improving gene editing efficiency in potato. To this end, the employment of an inactive dCas9 variant to fuse to the CTS-containing donor (Nguyen et al., 2020), while maintaining the RNP complex targeting the desired gene free of any interactions previous to the transfection, could be a strategy to test.

## 4 Conclusion

In this study, we explored conditions that have potential to favour HDR-mediated gene editing in potato. The employed combination of protoplasts with NGS offers an effective platform for rapidly assessing gene editing components and conditions for downstream applications. One main bottleneck of this platform is the isolation of high-quality protoplasts, typically considered labour-intensive. However, simplifying and optimising this step in potato and other plant species would promote broader use of this approach for validating gene editing tools. Our study on how HDR efficiency is influenced by the DRT structure, indicate that ssDNA donors matching the CRISPR/Cas9 target strand may be generally more efficient than other configurations, providing insights for future genome editing strategies in this species. Additionally, short ssDNA donors are prone to high-frequency insertions in the potato genome, mediated by alternative mechanisms other than HDR. While this may be a limitation of their application to precise modifications of coding sequences, this approach holds potential for efficient targeted insertions of cis-regulatory elements to modulate target gene expression in potato. Recent identification of short cis-regulatory elements in this species (Zeng et al., 2019; Wan et al., 2024; Zhu et al., 2024) coupled with efficient CRISPR/Cas9-mediated targeted insertion approaches, would contribute to fine-tune gene expression as a key driver of phenotypic novelty.

## 5 Materials and methods

### 5.1 Plant material

Potato cultivars Kuras (https://www.europotato.org/varieties/view/Kuras-E) and Desiree (https://www.europotato.org/varieties/view/Desiree-E) were used for protoplast isolation. *In vitro* plants were grown at 22 °C/18 °C (light/dark) under a photoperiod of 16 h light (120–140 μE m^−2^ s^−1^) and 8 h dark. Plants were propagated in sterile polystyrene containers RA85 (SacO2, Deinze, Belgium), each containing 75–80 mL of ½ × MS30 medium (½ × Murashige and Skoog salts and vitamins, 3% w/v sucrose, and 0.6% w/v Phyto Agar), supplemented with 8 µM silver thiosulphate (STS), adjusted to pH 5.8.

### 5.2 sgRNA design and RNP formulation

We identified target sites in sequences of the *soluble starch synthase 1 (SS1)*, *empfindlicher im dunkelroten licht 1 (EID1)*, *night light–inducible and clock-regulated gene 2 (LNK2)*, and *suppressor of SP6A expression (SES)* genes. Gene/locus accessions are listed in Supplementary Table S2. Target identification was performed using Cas-Designer (http://www.rgenome.net/cas-designer) as described (González et al., 2023). Complementarily, sgRNA efficiency and specificity were predicted using CRISPOR software (Concordet and Haeussler, 2018). For each target gene, sequences were retrieved from data of the doubled monoploid *S. tuberosum* Group Phureja DM1-3 516 R44 v6.1 (https://spuddb.uga.edu/).

For *SS1*, four target sites (T7, T12, T13, and T17; Figure 1A) were selected, and allelic variations were assessed using in-house genomic data of Kuras. For *EID1*, *LNK2*, and *SES*, two target sites per gene were selected (Supplementary Table S2). Sequence confirmation in Desiree was performed for *EID1* and *LNK2*, via PCR amplification and Sanger sequencing (oligonucleotides provided in Supplementary Table S3).

sgRNAs were purchased as unmodified synthetic RNA (Synthego, Redwood City, CA, United States). Prior to transfection, sgRNAs were resuspended in RNAse free water to 100 pmol/μL. RNP complexes were assembled using 100 pmol sgRNA and 30 pmol TrueCut Cas9 Protein v2 (Thermo Fisher Scientific) as previously described (Andersson et al., 2018).

### 5.3 Donor repair template (DRT) design

DRTs were designed based on the predicted Cas9 cut sites. The 5′ and 3′ homology arms (HAs) were derived from sequences located upstream and downstream of the DSB, respectively. In cases of allelic variation, a consensus sequence was used. A *BamHI* restriction site (5′-GGATCC-3′) was inserted into all DRTs after verifying its absence in target and flanking regions.

Single-stranded DNA (ssDNA) templates were synthesized as Ultramer Oligonucleotides (Integrated DNA Technologies, Inc.) at a 4 nmol scale, containing two phosphorothioate bonds located at the ultimate and penultimate linkages at both 5′ and 3′ ends (Schubert et al., 2021). ssDNAs were resuspended in sterile TE buffer (10 mM Tris, 0.1 mM EDTA, pH 8.0) to 100 pmol/μL and stored at −20 °C, when not immediately used. For transfections, 150 pmol DRT was added to 100,000 protoplasts immediately before RNP addition.

Double-stranded DNA (dsDNA) templates were obtained by annealing complementary ssDNAs at equimolar concentrations in TE buffer supplemented with 50 mM NaCl. Annealing was performed with a thermocycler program: 2 min at 95 °C, followed by 70 touchdown cycles of 30 s cooling from 95 °C to 25 °C (−1 °C/cycle), and a final hold at 4 °C. For transfections, 150 pmol of the annealed DRT was added to 100,000 protoplasts.

DRTs containing truncated Cas9 target sites (CTS) were designed as described (Nguyen et al., 2020; Shy et al., 2023). Briefly, truncated 16-nt sequence of the T17 site, along with the PAM (PAM-in orientation), was included at each DRT end, complemented by four mismatching nucleotides and extensions of 16 nucleotides from the HAs (Figure 4A). For the CTS-ds30 donor, complementary ssDNA sequences were synthesized as Ultramer DNA Oligonucleotides and annealed, as explained before. For CTS-ssT30, complementary oligonucleotides covering the PAM and truncated sites were annealed to the target strand ssDNA, creating short dsDNA regions at the ends. In all cases, 150 pmol DRT was mixed with RNPs, incubated for 5 min at room temperature, and subsequently added to protoplasts.

### 5.4 Protoplast isolation, transfection and culture

Protoplasts were isolated from 5-week-old *in vitro* plants as previously described (Nicolia et al., 2021), with slight modifications. After filtration through 100 μm and 70 μm cell strainers, suspensions were centrifuged at 70 × g (minimal acceleration/deceleration) for 10 min. Pellets were gently resuspended in 8 mL wash solution, and sucrose solution was added carefully beneath the suspension, using a sterile glass Pasteur pipette. After centrifugation at 70 × g for 20 min, viable protoplasts were collected from the interface.

All transfections were performed in triplicate for each treatment. One hundred thousand protoplasts were transferred to 15 mL centrifuge tubes already containing RNPs and DRTs (when applicable) and treated with 40% PEG solution (40% m/v PEG 4000, 73 g/L mannitol, 24 g/L Ca (NO_3_)_2_·4H_2_O) for 30 min. Transfections were stopped with 5 mL wash solution, and protoplast were centrifuged and resuspended in 1 mL Medium E (Nicolia et al., 2021). Protoplasts were cultured in static conditions at 24 °C in the dark.

For treatments with NHEJ inhibitors, compounds were added to Medium E prior to protoplast resuspension. Trichostatin A (Merck, Germany) was used at final concentrations of 0.01, 0.05, and 0.1 µM NU7441 (DNA-PK inhibitor; MedChemExpress, NJ, United States) was used at 0.5, 1, and 5 µM. HDR Enhancer v.2 (Integrated DNA Technologies, Inc.) was applied at 1 µM as per the manufacturer’s recommendation.

### 5.5 Next-generation sequencing (NGS) analysis

After 48 h culture, protoplasts were collected by centrifugation at 1,000 × g for 5 min at room temperature. Pellets were resuspended in 20 µL DNase-free water and incubated at 95 °C for 5 min. Aliquots (3 µL) were directly used for PCR amplification using Phusion High-Fidelity DNA Polymerase (Thermo Fisher Scientific) in 50 µL reactions, following the manufacturer’s instructions. Oligonucleotides for each target are listed in Supplementary Table S3.

PCR products were analysed by agarose gel electrophoresis, purified with the GeneJET PCR Purification Kit (Thermo Fisher Scientific), and quantified using a microvolume spectrophotometer. Purified amplicons representing biological triplicates from each experiment, were sequenced via Illumina MiSeq paired-end amplicon sequencing at Eurofins Genomics (Ebersberg, Germany).

### 5.6 Targeted mutagenesis

To assess on-target cleavage efficiency, the frequency of targeted mutagenesis in transfected protoplasts was evaluated. Sequencing reads (.fastq.gz files) were processed with CRISPResso2 (https://crispresso2.pinellolab.org/submission), with the following parameters: minimum homology for alignment to an amplicon = 60%: centre of quantification window (relative to 3′ end of the provided sgRNA) = - 3; quantification window size (bp) = 1; plot window size (bp) = 30–40; minimum average read quality (phred33 scale) = 30 (Clement et al., 2019).

When applicable, allele-specific targeted mutagenesis was calculated using the following formula:

$$Targeted\;mutagenesis\;\left(\%\right)=\frac{Modified\;reads}{Reads\;matching\;target\;allele}\;x\;100$$

Here, “Modified reads” refers to the number of reads classified as “Modified” by CRISPResso2, after excluding those that show allelic variation within the quantification window. This correction was performed using the “Alleles_frequency_table_around_sgRNA.txt” file generated by CRISPResso2. “Reads matching target allele” represents the total number of reads in the input aligning to the specific target allele. This was determined by subtracting the number of reads corresponding to alternate alleles from the “Reads_aligned” value (i.e., total number of reads aligned after CRISPResso2 pre-processing). The count of alternate allele reads was obtained using a custom R script that screened the “Alleles_frequency_table.txt” output from CRISPResso2. Values used in calculations are available in Additional data file.

To analyse the incidence of microhomology-mediated end joining (MMEJ) in total targeted mutagenesis, the T17 target site was analysed with the microhomology predictor available in CRISPR RGEN Tools (http://www.rgenome.net/mich-calculator/) (Bae et al., 2014). The top ten-ranked mutational outcomes (Supplementary Table S1) were searched with a custom R script that screened the “Alleles_frequency_table.txt” output from CRISPResso2.

### 5.7 Homology-directed repair (HDR) and targeted insertion frequencies

For experiments involving DRTs, CRISPResso2 analyses were conducted using the same parameters as previously described, providing both reference and expected HDR amplicon sequences (Clement et al., 2019).

HDR frequency was calculated using the formula:

$$HDR\;\left(\%\right)=\frac{HDR\;Reads}{Reads\_aligned}\;x\;100$$

Here, “HDR Reads” is defined empirically as the number of reads containing the *BamHI* restriction site insertion, along with scar-free recombination of both homology arms and the adjacent 5 bp flanking regions. This value was obtained using an R script that screened the “Alleles_frequency_table.txt” output file from CRISPResso2. “Reads_aligned” indicates the total number of reads contained in the “Alleles_frequency_table.txt” file. The values used for these calculations are provided in Additional data file.

Targeted insertion frequency was calculated using the formula:

$$Targeted\;insertion\;\left(\%\right)=\frac{Insert\;reads}{Reads\_aligned}\;x\;100$$

“Insert reads” denotes the total number of reads containing the *BamHI* recognition site insertion, as determined using the same R script.

### 5.8 Statistical analysis and graphics

Experimental data were analysed using Prism v8.0.1 (GraphPad Software, United States of America). Statistical significance was evaluated using one-way or two-way ANOVA (p < 0.05). When significant differences were observed, multiple comparisons were performed using either Tukey’s test (for comparing all group means) or Sidak’s test (for comparing each group mean with a control), both with a significance threshold of p < 0.05.

## Data Availability

The original contributions presented in the study are included in the article/Supplementary Material, further inquiries can be directed to the corresponding authors.
