# Supplementary material for: Single-stranded DNA (ssDNA) donor repair templates and CRISPR/Cas9 enable a high-frequency of targeted insertions in potato
Source: Front Genome Ed. 2025 Sep 19;7:1661829. doi: 10.3389/fgeed.2025.1661829 (PMC12491282; doi:10.3389/fgeed.2025.1661829)
Supplement: Supplementary file 1 [file Supplementaryfile1.docx]

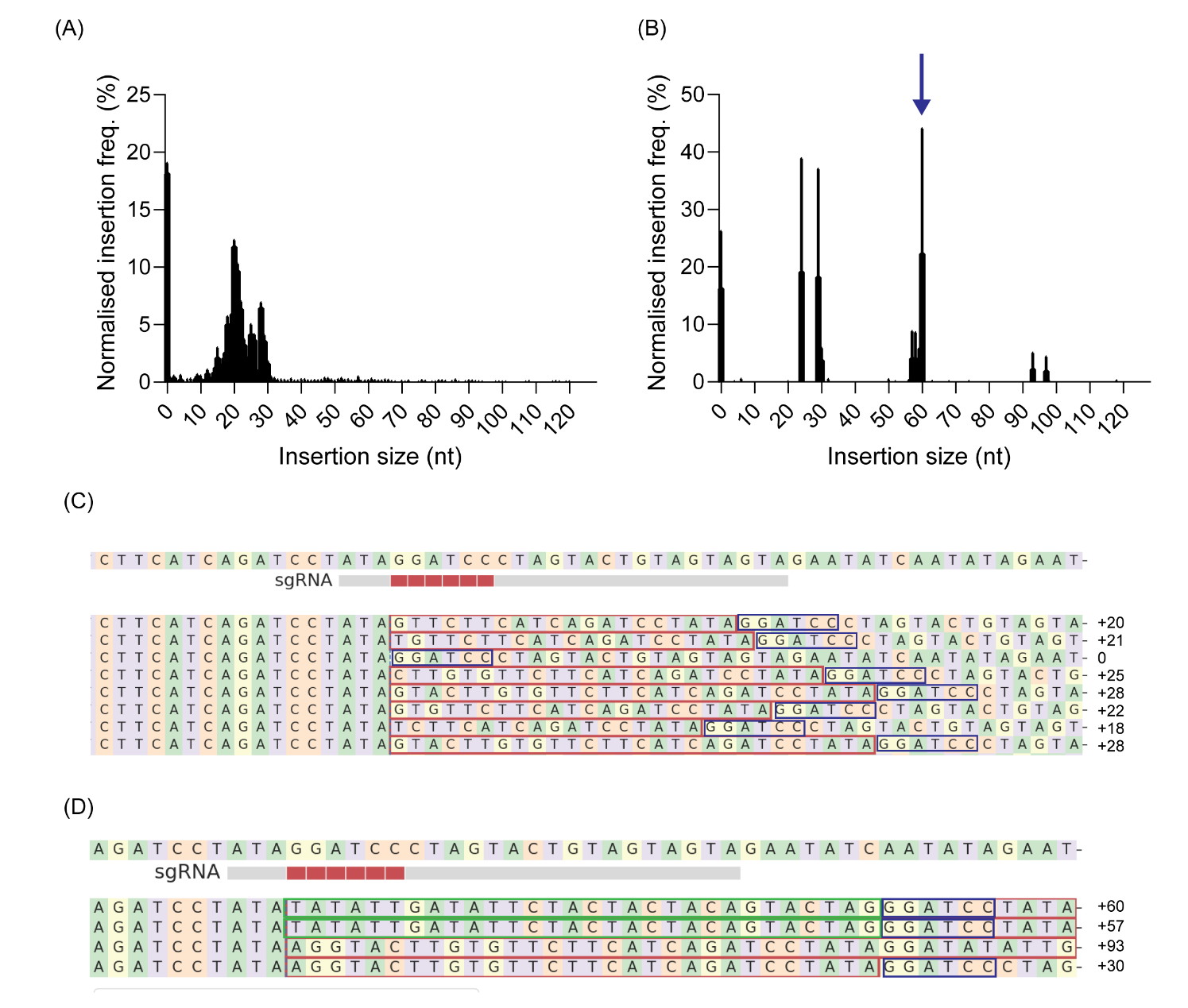
Supplementary Material

**Figure S1.** **Size distribution and sequence analysis of targeted insertions at the T17 target site. (A)** Distribution of normalised frequencies of various size insertions using a single-stranded DNA donor matching the target strand (ss-T donor) with 30-nt homology arms. **(B)** Distribution of normalised frequencies of insertions using a double-stranded DNA donor (dsDNA donor) with 30-nt homology arms. The blue arrow highlights a prominent peak at 60-nt insertions, consistent with complete donor integration and duplication of both homology arms. Normalised frequencies were calculated by dividing the number of reads with each insertion size by the total number of reads containing any insertion. Values represent the mean ± standard deviation from three biological replicates. **(C)** Example of CRISPResso sequence alignment of insertions from ss-T donor. Partial duplications of the 5′ HA are highlighted with red rectangles, and the inserted *BamHI* recognition sequence (5´-GGATCC-3´) is marked with a blue rectangle. The total number of inserted nucleotides is indicated for each sequence; “0” denotes perfect HDR event without duplication of flanking sequences. **(D)** Example of CRISPResso sequence alignment of insertions from dsDNA donor. Complete integration of the 3′-HA is highlighted with green rectangles (first and second rows), indicating reversely oriented insertions. Complete integration of the 5′-HA is shown with red rectangles (third and fourth rows). The *BamHI* recognition sequence is marked in blue. Total inserted nucleotide counts are shown for each sequence. Due to the maximum plot window size setting in CRISPResso, insertions longer than 40 nt are not visible in the alignment.

**
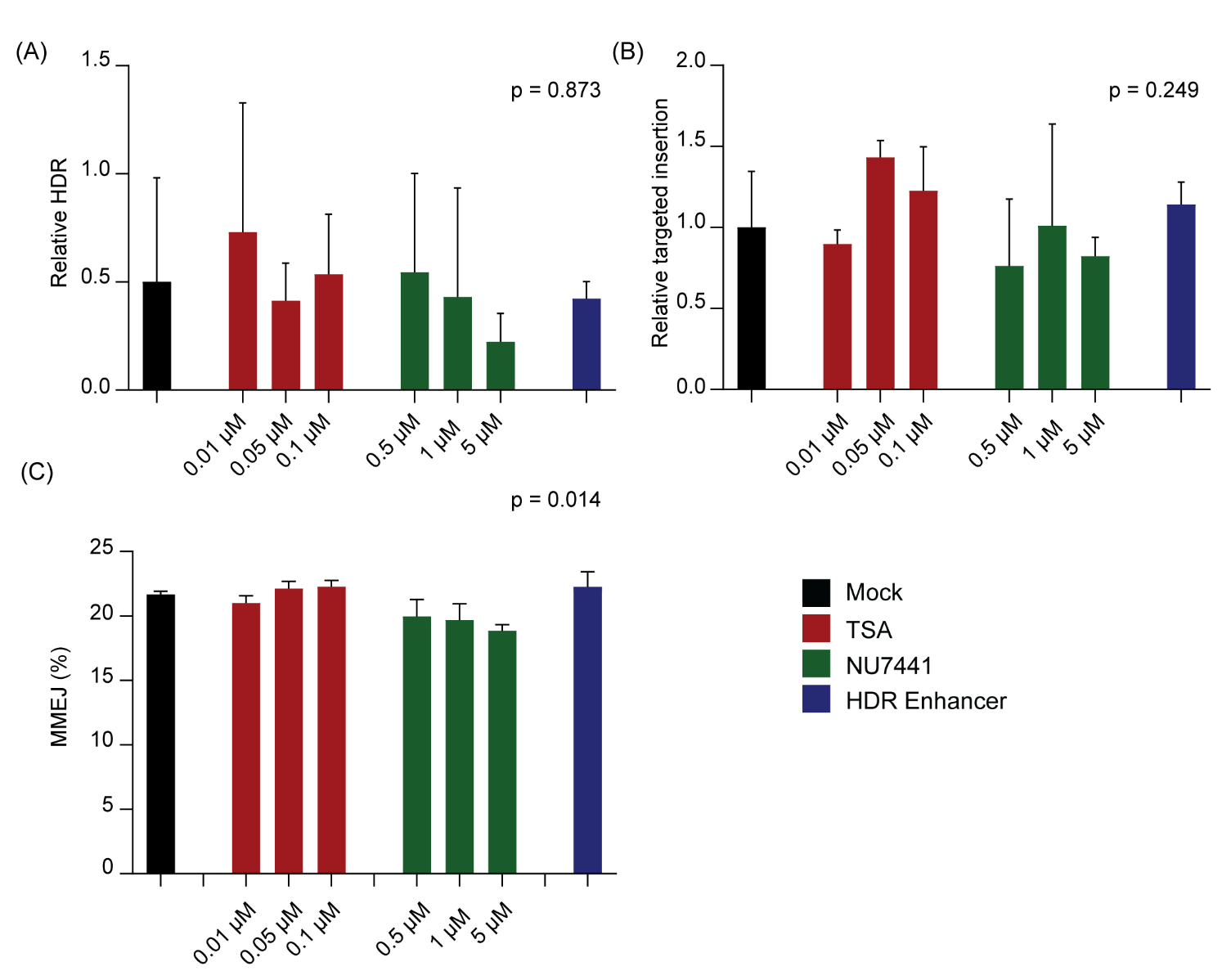
**

**Figure S2.** **Use of NHEJ inhibitors in potato.** **(A)** Effect of Trichostatin A (TSA), NU7441, and Alt-R HDR Enhancer on HDR efficiency at the T17 target site. **(B)** Effects of TSA, NU7441, and Alt-R HDR Enhancer on targeted insertions at the T17 target site. Data is presented as the mean of three independent biological replicates, with SD error bars. Values were normalized to the mean of the mock control. ANOVA’s p-value is indicated. **(C)** Effects of TSA, NU7441, and Alt-R HDR Enhancer on microhomology-mediated end joining (MMEJ) at the T17 target site. Data are expressed as the combined percentage of total modified reads matching MMEJ-predicted patterns, as listed in Table S1. Bars represent the mean of three biological replicates, and whiskers represent the standard deviation. The ANOVA p-value is indicated; no statistically significant differences were found between treatments and the mock in a post hoc Dunn’s test (p < 0.05).


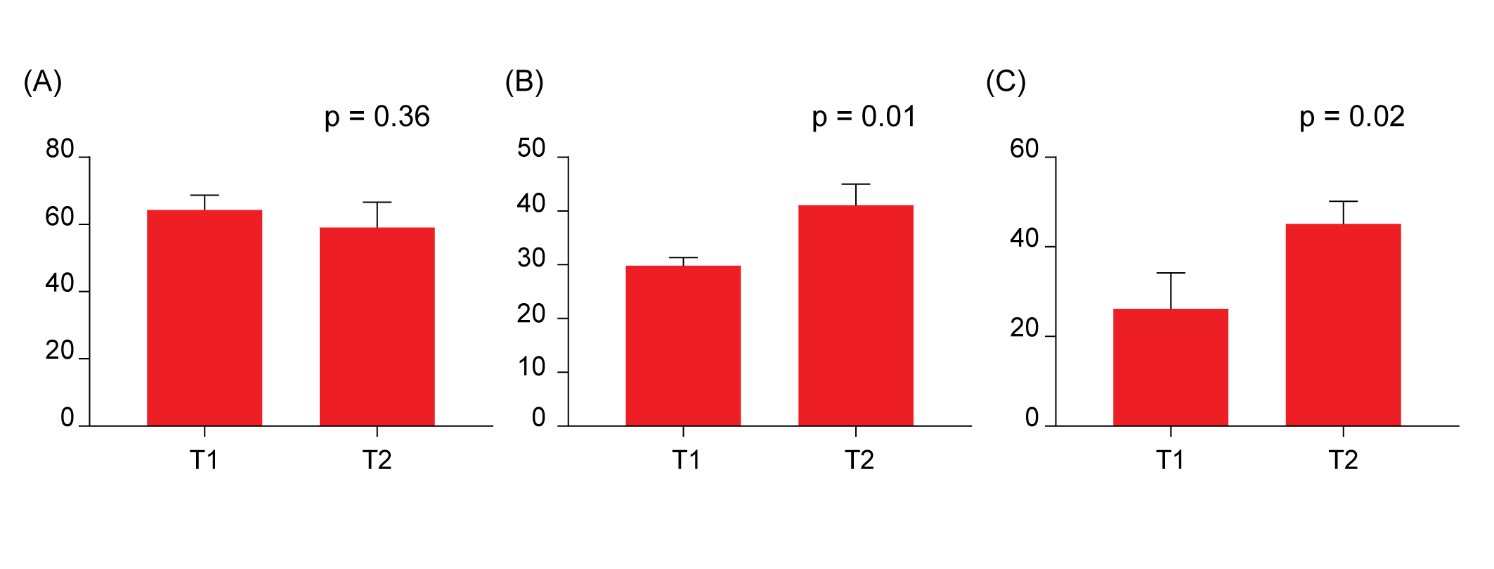
**Figure S3. On-target cleavage efficiencies.** Targeted mutagenesis (%) at two distinct target sites (T1 and T2) was assessed for each of the following genes: *EID1* **(A)**, *LNK2* **(B)**, and *SES* **(C)**. Data are presented as the mean of three independent biological replicates, with error bars representing the standard deviation (SD). An unpaired t-test was performed, and p-values are indicated on each graph. For *EID1*, the sgRNA targeting T1 was selected for further analysis. For *LNK2* and *SES*, the most efficient sgRNAs targeting T2 were chosen, respectively.

**
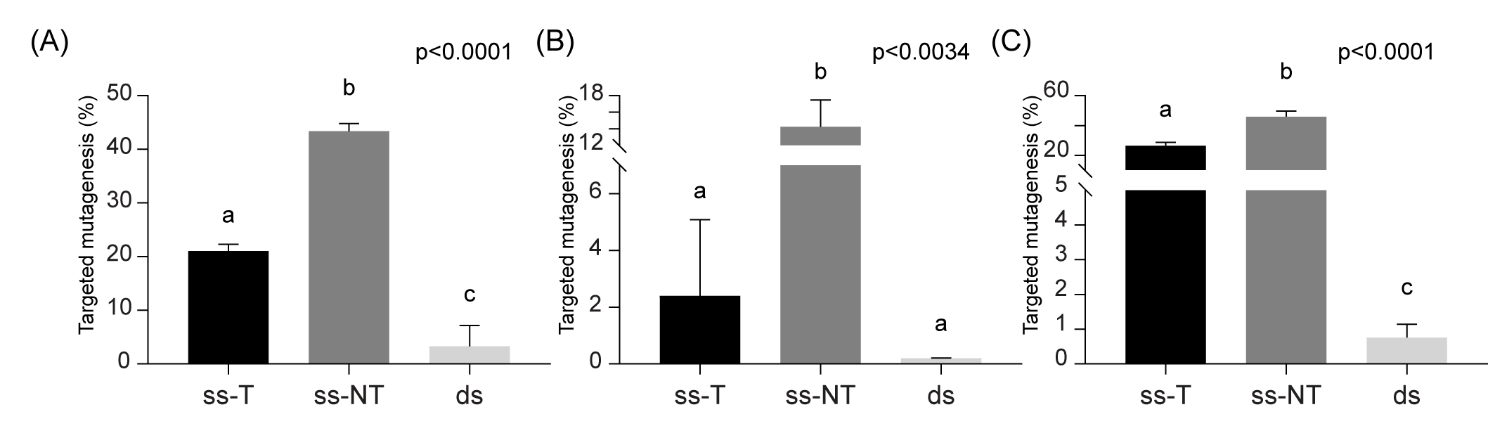
**

**Figure S4.** **Targeted mutagenesis** **(%) at the *EID1* (A), *LNK2* (B), and *SES* (C)** target genes, obtained in transfections with different DRTs. Data is presented as the mean of three independent biological replicates, and error bars indicate SD. ANOVA’s p values are indicated on each graph and different letters denote statistically significant differences determined using Tukey's multiple comparisons test (p < 0.05). “ss-T”, “ss-NT”, and “ds” indicate ssDNA in the target orientation, ssDNA in the non-target orientation, and dsDNA, respectively.

**Table S1 – Prevalence of microhomology mediated-end joining (MMEJ) in targeted mutagenesis on T17**

| MMEJ | Predicted pattern ^a^ | Deletion (bp) | Score | #Reads ^b^ |
| --- | --- | --- | --- | --- |
| WT | GTATCTGTCCACCTCTTTCTAGGTACTTGTGTTCTTCATCAGATCCTATACTAGTACTGTAGTAGTAGAATATCAATATAGAATGAGTGTTTCATTTTCA | n/a | n/a | n/a |
| 1 | GTATCTGTCCACCTCTTTCTAGGTACTTGTGTTCTTCATCAGATCCTA-----GTACTGTAGTAGTAGAATATCAATATAGAATGAGTGTTTCATTTTCA | 5 | 311.6 | R1: 228 R2: 84 R3: 159 |
| 2 | GTATCTGTCCACCTCTTTCTAGGTACTTGTGTTCTTCATCAGATCCT-----------GTAGTAGTAGAATATCAATATAGAATGAGTGTTTCATTTTCA | 11 | 173.1 | R1: 76 R2: 76 R3: 26 |
| 3 | GTATCTGTCCACCTCTTTCTAGGTACTTGTGTTCTTCATCAGATCCTATA---GTACTGTAGTAGTAGAATATCAATATAGAATGAGTGTTTCATTTTCA | 3 | 172.2 | R1: 7,056 R2: 6,531 R3: 6,667 |
| 4 | GTATCTGTCCACCTCTTTCTAGGTACTTGTGTTCTTCATCAG------------TACTGTAGTAGTAGAATATCAATATAGAATGAGTGTTTCATTTTCA | 12 | 164.7 | R1: 30 R2: 22 R3: 0 |
| 5 | GTATCTGTCCACCTCTTTCTAGGTACT-------------------------------GTAGTAGTAGAATATCAATATAGAATGAGTGTTTCATTTTCA | 31 | 148.4 | R1: 0 R2: 0 R3: 0 |
| 6 | GTATCTGTCCACCTCTTTCTAGGTACTTGTGTTCTTCATCAGATCCTATA------CTGTAGTAGTAGAATATCAATATAGAATGAGTGTTTCATTTTCA | 6 | 148.2 | R1: 3,175 R2: 3,324 R3: 3,078 |
| 7 | GTATCTGTCCACCTCTTTCTAGGTACTTGTGTTCTTCATCAGATCCTA--------CTGTAGTAGTAGAATATCAATATAGAATGAGTGTTTCATTTTCA | 8 | 134.0 | R1: 90 R2: 70 R3: 145 |
| 8 | GTATCTGTCCACCTCTTTCTAGGTACTTGTGTTCT-----------------AGTACTGTAGTAGTAGAATATCAATATAGAATGAGTGTTTCATTTTCA | 17 | 128.1 | R1: 0 R2: 0 R3: 0 |
| 9 | GTATCTGTCCACCTCTTTCTAG--------------------------------TACTGTAGTAGTAGAATATCAATATAGAATGAGTGTTTCATTTTCA | 32 | 121.2 | R1: 0 R2: 27 R3: 47 |
| 10 | GTATCTGTCCACCTCTTTCTAGGTACTTGTGTTCTTCATCAGATCCTATA-----------GTAGTAGAATATCAATATAGAATGAGTGTTTCATTTTCA | 11 | 115.3 | R1: 375 R2: 270 R3: 290 |
| **Total ^c^** | R1: 11,030 (51,212; **21.53%**)  R2: 10,404 (48,350; **21.51%**)  R3: 10,412 (47,381; **21.97%**) | | | |

a. Score of each pattern according to the microhomology size and the deletion length, as predicted by Microhomology-Predictor (<http://www.rgenome.net/mich-calculator/>). Microhomologies are indicated as red letters.

b. Number of reads matching each MMEJ-predicted pattern found per biological replicate (R1-R3).

c. Sum of total reads compatible with MMEJ mechanism per biological replicate. Numbers in parenthesis indicate the total number of reads with targeted mutagenesis; and the corresponding percentage explained by the identified MMEJ (bold).

**Table S2. Target loci and target sites**

| Target gene | Gene/Locus | Target site | Target sequence |
| --- | --- | --- | --- |
| SS1 | Soltu.DM.03G022350.2 | T7 | CACAAGTACCTAGAAAGAGG |
|  |  | T12 | GAACACAAGTACCTAGAAAG |
|  |  | T13 | ACGACATCGTTTTAGTATTT |
|  |  | T17 | TACTACTACAGTACTAGTAT |
| EID1 | Soltu.DM.09G023440.1 | T1 | GTATGGAGGACGATTAATGA |
|  |  | T2 | GGACGATTAATGATGGCAAT |
| LNK2 | Soltu.DM.01G025720.2 | T1 | GAAGGGAAAGCTCCAGCTTA |
|  |  | T2 | CTACTATGAAAGCTGGATGC |
| SES | chr03: 36702470..36702808, minus strand | T1 | ATAGTTGTGTTATGTTGTTT |
|  |  | T2 | ATATTCTTAATATGGTGTGA |

**Table S3. Oligonucleotides used in this study**

| Oligonucleotide ID | Sequence ^a^ | Description |
| --- | --- | --- |
| StSSI_F2NGS | ACACTCTTTCCCTACACGACGCTCTTCCGATCTGAGGTGACCGGTTAGATTTG | Amplification of *SS1* region spanning target sites T7, T12, T13, and T17, for Illumina MiSeq paired-end amplicon sequencing |
| StSSI_R2NGS | GACTGGAGTTCAGACGTGTGCTCTTCCGATCTCTCTCCCTGACACACATAAAC |  |
| EID1F | ACACTCTTTCCCTACACGACGCTCTTCCGATCTGTACATATGTGATTGGCCTGGT | Amplification of *EID1* region spanning target sites T1 and T2 for Illumina MiSeq paired-end amplicon sequencing |
| EID1R | GACTGGAGTTCAGACGTGTGCTCTTCCGATCTCCAATCAGTCCATGCTCCAGA |  |
| LNK2F | ACACTCTTTCCCTACACGACGCTCTTCCGATCTGGGCGAGACTGGTGAGAG | Amplification of *LNK2* region spanning target sites T1 and T2 for Illumina MiSeq paired-end amplicon sequencing |
| LNK2R | GACTGGAGTTCAGACGTGTGCTCTTCCGATCTCAGCACCCGAGGAATCTTGA |  |
| SESF | ACACTCTTTCCCTACACGACGCTCTTCCGATCTGCAAAATGATGTTCTAACAAAGG | Amplification of *SES* region spanning target sites T1 and T2 for Illumina MiSeq paired-end amplicon sequencing |
| SESR | GACTGGAGTTCAGACGTGTGCTCTTCCGATCTTTGAGAGTAACCCTTCTGAGT |  |
| EID1F-seq | TGGAATTTGAGCCGTGAGCA | Amplification and Sanger sequencing of *EID1* in Desirée |
| EID1R-seq | CCCAAAACAGCCTAAAATGGC |  |
| LNK2F-seq | TCCTGTTCCACCATTAGTTGGGC | Amplification and Sanger sequencing of *LNK2* in Desirée |
| LNK2R-seq | TTTCATGCAAAAGGCTGGAAACA |  |

1. Adaptor sequences for Illumina MiSeq sequencing analysis are underlined.
